# Barriers and Facilitators of Access and Adherence to Antiretroviral Therapy Among Young People Living with HIV in Shinyanga Region, Tanzania

**DOI:** 10.1007/s10461-026-05052-2

**Published:** 2026-02-03

**Authors:** Constantine Alex Ntanguligwa, Marta Tilli, Beatrice Borchi, Optat Kajuna, Angela Munishi, Christina Jahari, Chrispin Mgute, Idd Amiri Salehe, Marianne Strohmeyer, Valentina Petrini, Chiara Didonè, Giovanni Putoto, Gaetano Azzimonti, Alessandro Bartoloni, Lorenzo Zammarchi, Giulia Morigoni, Francesca Ierardi

**Affiliations:** 1 Doctors with Africa CUAMM, Shinyanga, Tanzania; 2 Department of Experimental and Clinical Medicine, University of Florence, Florence, Italy; 3 Infectious and Tropical Diseases Unit, Careggi University Hospital, Florence, Italy; 4 Doctors with Africa CUAMM, Padua, Italy; 5 Tuscany Regional Health Agency, Florence, Italy

**Keywords:** Access to HIV services, Adherence to ART, Adolescents and young adults living with HIV, Barriers, Facilitators, Tanzania

## Abstract

Despite advancements in antiretroviral therapy (ART) in Tanzania, adherence remains a challenge, especially among young people living with HIV (YPLHIV) aged 10–24 years. This study, conducted in the Shinyanga region of Tanzania, explored the factors affecting access and adherence to ART among YPLHIV. Access to ART refers to the ability to reach, enroll, and receive HIV care and education; adherence refers to proper use of medications as prescribed. The research employed focus group discussions and in-depth interviews with YPLHIV and their caregivers. Data were transcribed and thematically analyzed using Atlas.ti software. The qualitative data analysis revealed clear categories of barriers and facilitators. Barriers to ART access and adherence included forgetfulness, lack of privacy, family instability, poverty, reflected in lack of food or water. Perceived stigma and discrimination from family, school or community members played a significant role, alongside low participation in adherence support activities. Factors facilitating ART access and adherence included disclosure of HIV status and access to social or emotional support from the family members, peers, teachers, health workers, and community members. These factors may increase knowledge about the risks of treatment failure and drug resistance, help reduce their perceived stigma and discrimination and support treatment continuity. Interventions by health workers, family members, peers, teachers—at individual and social levels—as well as by government and local health authorities—at the policy level—are needed to address the above barriers and facilitators to ART access and adherence among YPLHIV enrolled in HIV care. A pesar de los avances en la terapia antirretroviral (TAR) en Tanzania, la adherencia sigue siendo un desafío, especialmente entre los jóvenes que viven con VIH (JVVHIV) de entre 10 y 24 años. Este estudio, realizado en la región de Shinyanga en Tanzania, exploró los factores que afectan el acceso y la adherencia a la TAR entre los JVVHIV. El acceso a la TAR se refiere a la capacidad de llegar, inscribirse y recibir atención y educación sobre el VIH; la adherencia se refiere al uso adecuado de los medicamentos según lo prescrito. La investigación empleó discusiones en grupos focales y entrevistas en profundidad con los JVVHIV y sus cuidadores. Los datos fueron transcritos y analizados temáticamente utilizando el software Atlas.ti. El análisis cualitativo reveló categorías claras de barreras y facilitadores. Las barreras al acceso y la adherencia a la TAR incluyeron el olvido, la falta de privacidad, la inestabilidad familiar y la pobreza, reflejada en la falta de alimentos o agua. La percepción de estigma y discriminación por parte de familiares, compañeros de escuela o miembros de la comunidad desempeñó un papel significativo, junto con una baja participación en actividades de apoyo a la adherencia. Los factores que facilitan el acceso y la adherencia a la TAR incluyeron la divulgación del estado serológico y el acceso a apoyo social o emocional por parte de familiares, compañeros, maestros, trabajadores de la salud y miembros de la comunidad. Estos factores pueden aumentar el conocimiento sobre los riesgos de fracaso del tratamiento y resistencia a los medicamentos, ayudar a reducir la percepción de estigma y discriminación y apoyar la continuidad del tratamiento. Se necesitan intervenciones por parte de los trabajadores de la salud, familiares, compañeros, maestros—a nivel individual y social—así como por parte del gobierno y las autoridades sanitarias locales—a nivel de políticas—para abordar las barreras y facilitadores mencionados en el acceso y adherencia a la TAR entre los JVVHIV inscritos en cuidados por VIH.

## Introduction

 Every day, approximately 4.000 new HIV infections occur worldwide, 60% of them reported in Sub-Saharan Africa [1]. In this region, about 8 out of 10 adolescents living with HIV [2–6].

Over the past two decades, the global response to HIV/AIDS has recorded a marked decline in AIDS-related infections and deaths, including among adolescents and young adults [7]. Nevertheless, substantial variability persists across countries.

Persistent new HIV infections, especially among youth, remain a major public health challenge across several East and Southern African countries, including Tanzania. Therefore, improving access to and adherence to antiretroviral therapy (ART), for young people aged 10–24 years as a whole and those living with HIV (YPLHIV), remains essential to ensure their physical, mental and sexual well-being. At this age, the majority of young people are in a vulnerable developmental stage associated with prioritisation of peer relationships, increased child-parent conflicts, and increased emotional and hormonal drive to explore their sexuality [7, 8]. Furthermore, the difficulty in accessing ART has a negative impact on physical well-being due to poor adherence to ART, which can lead to treatment failure, the emergence of drug resistance and HIV transmissions [2]. Adequate access to ART remains a key issue for this age group. As reported by the Tanzania HIV Impact Survey (THIS) 2016–2017 [9], young people are less likely to access to ART, which has implications for HIV surveillance and control. Similar observations have been reported in some studies that young people aged 15–24 years are less likely to be enrolled in ART and HIV care, and also less likely to adhere to ART [3]. Efforts to investigate the situation of barriers and facilitators affecting access to and adherence to ART among YPLHIV have been conducted across several sites. Whereas the 2016–2017 Tanzania HIV Impact Survey indicated that discriminatory attitudes toward YPLHIV were most prevalent among people in rural and semi-urban areas [9], nearly all studies about access and adherence to ART in Tanzania were conducted in urban settings and predominantly focused on the adults. A significant knowledge gap exists regarding the factors affecting access and adherence to ART among YPLHIV. Therefore, this study investigated the factors that hinder and facilitate access to and adherence to ART among YPLHIV and their guardians in Shinyanga region in Tanzania, to provide evidence for devising interventions that enhance ART access and adherence to particularly among YPLHIV who reside in rural and semi-urban areas of the Shinyanga region. The study was guided by Bronfenbrenner’s social ecological model for human development [10], which asserts that human behaviour and development are affected by interactions between individuals and their surrounding environmental systems. These systems range from immediate relationships to broad cultural values. In this study ART access refers to the ability to reach, enroll for and receive HIV-related education, medical and psychosocial care, which goes beyond the availability of facilities providing HIV-related services such as testing and enrollment into ART care. ART adherence includes attending scheduled appointments and utilization of ART as prescribed; but this also includes the effective management of the medication supply chain, including receiving, storing and replenishing HIV medications in a timely manner to ensure an uninterrupted and maximally effective treatment course. Accordingly, barriers and facilitators refer, respectively, to obstacles and enablers, that affect ART access and adherence. The research was conducted by health professionals from the Tuscany Regional Health Agency (Quality and Equity Unit), Infectious and Tropical Department of Careggi University Hospital, Italy, and Doctors with Africa (Cuamm), an International non-governmental organisation, with the aim of identifying barriers and facilitators to access and adherence to antiretroviral therapy among young people aged 10 to 24, with guardians providing information for adolescents aged 10 to 14.

## Methods

### Study Design

This was a descriptive qualitative study, which used focus group discussions (FGDs) and in-depth interviews (IDIs) to collect data from at one guardian of YPLHIV, who were aged 10 to 14 years and YPLHIV aged 15 to 24 years, who attending HIV care and treatment at Ngokolo, Shinyanga, and Bugisi health facilities in Shinyanga district in Tanzania..

### Study Participants

The study population included YPLHIV aged 10–24 years receiving ART at the following Care and Treatment Clinics (CTCs): Shinyanga Regional Referral Hospital, Ngokolo Health Center (Shinyanga Municipal Council), and Bugisi Health Center (Shinyanga District Council). Young people aged 15–24 years were included because they can reliably report their own experiences with ART access and adherence, and this age group represents a critical stage of transition to autonomous management of HIV care. Guardians of adolescents aged 10–14 years were also included to provide reliable information on ART access and adherence, as younger adolescents may have limited ability to accurately report their medication use. Inclusion criteria were: age 10–24 years, residence in Shinyanga Municipal or District Council, on ART for at least 6 months. Exclusion criteria were: age below 10 or above 24 years, residence outside Shinyanga Municipal or District Council, on ART for less than 6 months. Non-Adherence was defined as self-reported missing one or more doses of ART over the past 30 days. Participants missing more than one dose during this period were classified as non-adherent. For younger adolescents, guardians provided reports of missed doses when required.

A total of 69 participants were recruited: 25 males (15–24 years), 22 females (15–24 years), and 22 guardians (10–14 years).

### Sampling Strategy

Eligible participants meeting the inclusion criteria were then stratified into three groups per facility to allow age-specific reporting: young women living with HIV (15–24 years), young men living with HIV (15–24 years), and guardians of adolescents (10–14 years). Simple random sampling strategies were used to select participants of this study. All eligible participants were assigned unique identification numbers, which were entered into an online randomization tool that generated a random list. Participants were then selected according to the randomly generated sequence until the required sample size for each group was reached. Selected participants were contacted by trained research assistants, provided study information, and invited to participate. Those who consented were assigned to the appropriate FGD. Participants who required further exploration of specific themes identified during the FGDs were subsequently selected for in depth interviews to gain more detailed insights.

### Data Collection Methods

Data for this study were collected using FGDs and IDIs with YPLHIV aged 15–24 years who were enrolled on ART and with the caregivers of younger adolescents aged 10–14 years who were enrolled on ART, to obtain a detailed understanding of the collective and personal factors influencing access and adherence to HIV treatment among these groups.

Altogether, nine FGDs (i.e., three sessions consisting of male YPLHIV aged 15–24 years, three sessions consisting of female YPLHIV aged 15–24 years, three sessions comprising caregivers of YPLHIV aged 10–14 years) and seven IDIs (i.e., six sessions with YPLHIV and one session with the caregiver of a younger adolescent who received ART) were conducted across the three selected health facilities. Each FGD session consisted of four to eleven participants (see Tables 1 and 2).

**Table 1:** Participants in FGDs, separated according to gender, age, and HIV care centre

Care and Treatment Centre	Type of participants			
	Caregivers of YPLHIV aged 10–14 years	Male YPLHIV aged 15–24 years	Female YPLHIV aged 15–24 years	Total
Regional Referral Hospital	8	9	11	28
Bugisi Health Centre	4	10	4	18
Ngokolo Health Centre	10	6	7	23
Total	22	25	22	69^a^

^a^Nine FGD sessions were conducted. Each FGD session included four to eleven participants

**Table 2:** Participants in IDIs, separated according to gender and HIV care centre

Care and Treatment Centre	Type of participants			
	Caregivers of YPLHIV aged 10–14 years	Male YPLHIV aged 15–24 years	Female YPLHIV aged 15–24 years	Total
Regional Referral Hospital	1	2	1	4
Bugisi Health Centre	–	2	–	2
Ngokolo Health Centre	–	–	1	1
Total	1^a^	4	2	7

^a^ Only one guardian agreed to participate in the in-depth interviews. This guardian was female

### Instruments

A set of unstructured questions were used to guide the FGDs and IDIs with the participants in this study. These questions were divided into three parts. The first part included questions about the participant’s age, gender, education status, employment status, housing and family situation, future aspirations, and experiences after discovering the personal HIV status.

The second part included questions related to the participant’s experiences about access and adherence to ART, such as, *when did you learn of your HIV status? When did you enrol on ART? Where did you enrol for ART? What have been your experiences with the service providers in the clinic you receive ART*,* the quality (i.e.*,* diversity*,* privacy and timeliness) of services in the clinic you receive ART*,* the support you receive from the different family members or community workers or social groups regarding your HIV treatment*,* when you disclosed your HIV status to others*,* or when others knew that you were on ART*.

The third part included questions that solicited the participant’s views about the specific situations in the family, school, healthcare and community that enable and deter them from accessing and adhering to ART, and the interventions for addressing them in these settings.

### Data Collection Procedures

The research was conducted by a multidisciplinary team consisting of 4 researchers who consisted of clinical and public health workers.

All team members received training in conducting focus group discussions and in depth-interviews, both as moderators and observers.

Team members were then assigned to conduct FGDs in one of the three hospital settings in such a way that groups with men and women were led by researchers of the same sex as the interviewees.

A targeted sampling approach was employed to recruit participants from the three facilities of Shinyanga Regional Referral Hospital, Ngokolo Health Center, and Bugisi Health Center. Clinic records were reviewed by trained research assistants to identify potential participants who met the inclusion criteria. Eligible patients were contacted by telephone, grouped into HIV-positive young males, young females and guardians of adolescents, and invited to attend the facility on a pre-arranged date for focus group discussions. On the scheduled day, all potential participants were convened and briefed collectively about the study’s objectives, ethical considerations, and procedures before recruitment into FGDs.

Participants were given full explanations of the objectives and procedures of the FGD and IDIs and it was specified that their participation was entirely voluntary.

For each participant, whether YPLHIV or their guardian, informed consent was obtained before participating in the project through the provision of a consent form, which also included permission for audio recording of the discussions and interviews. For participants under the age of 18, consent was obtained from their guardian.

Both the FGDs and IDIs were conducted in private rooms to ensure confidentiality.

FGD was facilitated by two team members fluent in the local language: a moderator who guided the discussion and an observer responsible for taking notes and monitoring group dynamics and non-verbal behavior. After introducing himself, the interviewer asked each participant to introduce themselves in turn. At the beginning of each FGD, he verbally reiterated the request for consent to participate and to record the session, assuring that a refusal to consent would carry no consequences. The interviewer then proceeded to read each question and allowed participants to respond one at a time, encouraging them to share their views with the group.

Subsequently, a subset of FGD participants was selected for further exploration of specific topics through in-depth interviews (IDIs) conducted by the same research team. Participants were selected by the psychologist based on key themes that emerged during the FGDs, particularly those related to narratives of physical or psychological violence within the family and community related to HIV status.

Data were collected in March 2023.

All qualitative data (collected through interviews and focus groups) were audio-recorded, transcribed, and translated into English. The focus group discussions lasted between 60 min and 90 min, while the interviews lasted between 9 and 15 min.

### Data Analysis

Data for this study were analysed using Grounded Theory, emphasizing social process and change over time. The Grounded Theory principles encourage researchers to investigate exhaustively the characteristics, conditions, causes, and antecedents in order to integrate all these elements into a theoretical framework [11, 12]. Data analysis have been conducted both manually and with the aid of Atlas.ti software (©Scientific Software Development GmbH) to facilitate the organization and management of the codes. Two researchers independently coded all transcripts, and any discrepancies were resolved through discussion until consensus was achieved to ensure coding reliability. Initially, broad thematic codes were identified to highlight the explanatory frameworks employed by participants. Subsequently, the researchers performed more detailed coding, focusing on specific elements of the illness experience. At each level of coding, discussions were held among researchers to reach consensus about the codes and their interpretations. In the first phase excerpts were categorized into two macro-categories: ‘Facilitators’ and ‘Barriers’ to HIV testing, access to care and the continuum of care as experienced by participants. In the second phase, more specific categories were identified within each macro-theme using broad codes. For the ‘Facilitators’ theme, the categories identified were: support and inclusion within the private/individual sphere and support and inclusion within the public sphere. For the ‘Barriers’ theme, sub-categories included psychosocial and emotional barriers on one hand, and practical and behavioural barriers on the other. Finally, in the third phase, each subcategory was further refined into specific categories, which received final labels. For facilitators, these were: family support and inclusion, community support and inclusion, health services support and good practice support. For barriers, the final categories were: fear of stigma and need for privacy, discrimination and, barriers to regular ART adherence.

An interpretive analysis contextualised these findings, linking them directly to the study’s objectives.

## Results

### Factors that Deterred YPLHIV from Accessing and Adhering to ART

Both FGDs and follow-up IDIs (*n* = 7) were included in the analysis. The IDIs confirmed the themes and patterns identified in the FGDs and did not reveal additional barriers or facilitators beyond those already reported.

(young people and guardians) reported several factors that hindered YPLHIV from accessing and adhering to ART. These included:

***Experience of stigma and discrimination from various sources***,*** including family members***,*** teachers***,*** friends***,*** neighbours and healthcare providers:*** A younger female participant who received HIV care from Y clinic said: “some people associate living with HIV with prostitution and sometimes say that I will die of HIV.” Another female participant who received HIV care from the same clinic said: “Once the teachers found out that you are living with HIV, they stigmatize you. This makes you feel bad because even those that did not know [about your HIV status] get to know that you are diseased. At school, most of my friends didn’t know my situation, but once they found out, they would gossip with others just to warn them that they shouldn’t sit or walk with me. Sometimes in the community you hear people discrediting you and some even say that you are about to die.” Similarly, a guardian of younger participant who received HIV care from the same Y clinic said: “… the society highly stigmatizes the children living with HIV because they are seen as less important compared to other children.”

These quotes show how social stigma and discrimination remain deeply rooted across multiple social levels—family, school, and community. Stigma arises from moral prejudice and misinformation, leading to exclusion, gossip and discrimination. Society tends to consider these young people less important. This causes isolation, shame and loss of self-esteem.

***Worry about the health workers***,*** family members***,*** school learners and teachers***,*** and other community members knowing of and disclosing their HIV status to others:*** Furthermore, a female respondent who received HIV care from the Y clinic reported: “I remember a nurse disclosing [informing me about my HIV care status] without privacy … when there were other people including my neighbors. At times I would hear the service providers [attributing] my HIV situation to prostitution and saying that [I] will die of HIV. You [I] cannot trust such a person … It is better to keep your HIV status a secret”. Another female respondent who received HIV care from the same clinic reported: “Whenever we receive visitors at home, I do not take the ART on time to avoid being seen, stigmatized or discriminated. By the time they leave, it is either too late or I would have forgotten to take the medicine”. Similarly, the guardian for a child who received HIV care from the Z clinic reported: “…when you take medicines from the clinic, the service provider sometimes asks you about the child’s progress, you explain everything hoping to receive help … only to realize that they disclose the information to the [streets] others. This is so hurting to [the child] us and the caregiver … it brings disappointments … Therefore, I recommend that there should be people [service providers] who are trained at the clinics … they could be given seminars to do their work well … such that the confidentiality is observed”. The guardian of another child who received HIV care from another X clinic also said: “It is not easy to tell teachers or classmates that this child is living with HIV, because then they will treat him as if he were infected”.

These quotes highlight how fear of involuntary disclosure and lack of confidentiality generate mistrust toward healthcare workers and teachers, leading to isolation, treatment interruptions and difficulties in ART accessing and adhering among YPLHIV and their guardians.

***Experience of judgmental and social distancing stigma from the family members***,*** school teachers and learners and community members:*** A young male who received HIV care from the Y clinic said: “Maybe at school, one of the teachers who knew my status told my other two female teachers. They always look at me in a way I feel bad. Psychologically it just affects me because of they talk about me. At school I don’t want people to know”.

This quote highlights how stigmatizing attitudes and social exclusion in schools and communities lead to psychological distress, feelings of isolation, lack of confidence, and resistance to disclosing HIV status, which can negatively impact treatment access and adherence among PLHIV.

***Lack of treatment support at home and school:*** A female respondent who received HIV care from the X clinic stated: “My father doesn’t want to see me at home, claiming that I might infect others. He prevents me from taking my medicine on time. I remember one day when he asked me to commit suicide. It hurt me.” Another female respondent who received HIV care from another facility, the Y clinic, also said: “Sometimes you [can] get sick during the exams, which prevents you from taking them. When you contact the teachers, most of them refuse to let you take the exam on another day, claiming that it isn’t their fault and that they have already finished marking.”

These quotes highlight a serious lack of support for PLHIV, both at home and at school. At home, stigma and discrimination hinder treatment and cause emotional harm; at school, the absence of understanding and flexibility leads to further exclusion. Overall, a crosscutting issue of marginalization and lack of support emerges, calling for integrated interventions to combat stigma and promote more inclusive environments.

***Family conflicts:*** On this topic, a woman interviewed by the HIV X clinic reported: “My father has a doctor friend who came home to see me, took me to the hospital and found that I had low hemoglobin. The doctor told my father that I should live with my biological mother or someone who would not stigmatize me. My father refused because he lives with his wife and has two wives who do not have good relationships with each other. The doctor suggested taking me to my aunt, but my father refused again, saying that my mother had left me with him and that he could not entrust me to anyone else”.

This quote - starting from the specific experience of the father’s refusal to provide support to his daughter, despite medical advice - more generally reveals how stigma, rejection, and family tensions interact to limit emotional support, ART access and adherence for PLHIV.

***Mistreatment from the caregivers and teachers:*** A young female living with HIV in care from Y clinic explained: “I was living with my stepmother. Whenever I made a small mistake, I was as being subjected to discriminatory abuse. When friends came to the house they were told not to play with me because I’m sick.” A male respondent receiving care from another facility, the X clinic, also stated: “My family knows [my HIV status], but I am scared of them, because they are harsh.”

These quotes highlight how fear of harsh reactions and discrimination from family members create barriers to emotional well-being and social inclusion, underscoring the crucial role of the family as a supportive and non-stigmatizing environment.

***The perceived lack of privacy in the HIV service centres:*** In the words of a female respondent from Y clinic is reported: “You might go to the HIV clinic and meet people you know. However, not everyone keeps this secret; they tell others, and so on.” A child guardian respondent from the same Y clinic said: “These clubs exist to provide a service to people with HIV. The main problem is that community members notice who goes there, assume that you are going there for treatment and therefore are living with HIV.”

These quotes highlight concerns about the lack of privacy in HIV care centers that YPLHIV and their guardians may have. The unwanted disclosure of HIV status, which contributes to stigma and social pressure, discourages their attendance at support centers and adherence to clinic visits.

***Perception that use of HIV medicines could deny them the opportunity to participate in some activities:*** A female participant from Y clinic explained: **“**I was very good at school, but when I realized I was stigmatized, I started to get discouraged and think that there was no point studying and taking medication. I thought that if I was stigmatized because of my HIV status, what is the point of me taking drugs now?”

This quote highlights the profound impact of social stigma on personal goals. Feeling judged or marginalized because of their HIV status can lead YPLHIV to undervalue treatment adherence and give little importance on daily responsibilities. This indicates how emotional and social factors can directly affect a young person’s ART adherence and participation in normal life activities, such as school attendance.

***Experience of psychosocial (psychological/emotional and relational/social) problems:*** A female respondent from one of the facilities, the X clinics, reported: “… whenever I am stigmatized, I feel worthless. If the stigma comes from family member, I feel even more frustrated and experience mental distress. Sometimes, I avoid participating in social activities and relationships. I feel I would never find someone to marry and feel like I don’t want to continue ART.”

This quote illustrates how stigma can deeply affect both mental health and social relationships. When discrimination comes from family members, feelings of worthlessness, social withdrawal, and doubts about ART adherence are intensified, highlighting the PLHIV’ linked psychological and relational challenges faced.

***Unawareness of the personal HIV status or not being notified about the HIV status:*** One female respondent attending X clinic shared: “I came to know about my status through my neighbors because they were stigmatizing me a lot, they would not let their children play with me telling them that I could infect them because I am HIV-positive.”

This quote underlines the negative impact of not being informed about one’s HIV status. The respondent learned of her status indirectly through neighbors, which exposed her to stigma and social exclusion, highlighting how lack of disclosure can worsen psychological and social challenges for young people living with HIV.

***Forgetting to swallow the medicine either due to much work at home and school or due to participation in several social events:*** A female respondent, receiving care at X clinics, reported: “I plan to take my ART every day at 8 p.m., but when the time comes, I’m not at home, so I can’t take it because I don’t have my medicine with me.” Similarly, another female from the same clinic noted: “During exams, I might sleep before 9 p.m., so that I can wake up at midnight to study again and my phone has no alarm and there is no one to remind me.” A male respondent receiving care at the same clinic reported “Sometimes I am playing football and I forget to take ART.”

These quotes showed that responsibilities and social activities can lead YPLHIV to forget their medications, highlighting how very busy daily routines and lack of reminders can undermine ART adherence.

***Poverty reflected by shortage of food to eat***,*** long distance to some HIV clinics and money for transport:*** A female respondent receiving care at X clinic observes: “I continued to live there, but sometimes I went hungry, sometimes I was not given bus fee to go to school so I had to walk from Ibuluma to Meko. After that, I asked my father to take me to school again so that I could continue my studies, but he said he has lack of money for transport.”

This quote highlights how poverty (specifically, food insecurity, long travel distances, and lack of funding for transportation) significantly hinders guardians’ ability to support young people’s access to ART and education, particularly in rural areas, and when weather conditions are challenging.

### Factors Enabling ART Access and Adherence Among YPLHIV

The participants (young people and caregivers) reported several factors that enabled ART access and adhere among YPLHIV. These included:

***Disclosure of the HIV situation to the family members***,*** teachers and significant others in the community:*** A young male being treated at Clinic Z reported, “I felt bad and thought about suicide because I heard two girls from my school teasing me about my father dying of HIV. I started telling my grandmother.” A caregiver of a child receiving treatment at Y clinics stated, “I shared the responsibility of caring for our brother’s son with a relative. We help each other. It would have been difficult alone. This problem isn’t just mine, so any family member could help.” Another caregiver of a child receiving treatment at the same clinic stated, “I shared because hiding isn’t a problem, but you choose whom to share with, based on the help they can offer—not necessarily financial, but also advice or ideas.” Similarly, Another caregiver of a child receiving treatment at X clinic said, “It’s good to inform the teachers so they’re aware; there’s transparency these days. If something happens, the teacher can call you. But if you hide from the teacher and something happens, you can blame yourself. So, it’s much better to tell the teacher because she is with the child from morning to night.” Sharing a child’s HIV status with neighbors can help caregivers both physically and emotionally when it comes to accessing and adhering to their child’s antiretroviral therapy.

This quote shows that sharing an adolescent or young adult’s HIV status with trusted family members, teachers, and neighbors provides both emotional and practical support, facilitating consistent access to and adherence to antiretroviral therapy. This support is especially important for guardians of child or adolescents, who may have limited understanding of the importance of ART continuity.

***Emotional and treatment support from the caregivers and other family members:*** A female receiving care at Y clinics reported: “Currently I reside in Mbeya. My mother comes to the clinic, takes my ART, packs it with some other small items and clothes she buys me, and sends everything together on the bus to me. Similarly, a child’s guardian from the same clinic said: “My child is a student, and there are times when he needs to come to the clinic, but he might be at school. Thus, I go to refill his ART for him so he then continues using the ART as long as he has no problem.” A young male receiving care at Clinic Z reported: “Grandma convinced me to stop thinking about being teased at school because my father died of HIV and asked me to focus on taking my medicine. So I stopped thinking about it.” A female recruited from X clinics reported: “My grandfather told me: ‘My granddaughter, look at how many people you meet when you go to the clinic for drug refills—they are healthy because they adhere to ART. You don’t have to be so hard on yourself.’” A child’s guardian from Y clinic highlighted “When it’s time, I remind him to take his medicine because he is still young and can’t take care of himself. He is still in the age where he can’t be careful, so as a parent, it is my duty to insist that he takes his medicine on time and to ask wether he has taken or not.”

These quotes hightlighted that family members play a crucial role in supporting YPLHIV in accessing and adhering to ART, providing both emotional encouragement and practical assistance, such as reminders or picking up medications. This support counteracts stigma and ensures consistent adherence. The quotes illustrate how such support is particularly important for children and adolescents, who may not fully understand the significance of treatment continuity.

***Emotional and treatment support from the teachers:*** A female interviewed from X clinic said: “I went to the principal and told him everything. He understood me, took my cards and set the date with the responsible health center so that when the date reaches arrived, I could go.” Another female from the same clinic reported: “My class teacher was the only one who knew, so it was easy to get permission for my dates of taking medicine or appointment for blood collection.” The guardian of a child receiving treatment at Y clinics said: “There is an elder in the village who helps us a lot with clinic care. Sometimes he calls me to remind me of things to do to improve my brother’s health… I call this elder and explain my difficulties because I know that I will not have money every day.”

These quotes suggest that support from trusted community members—such as teachers and village elders—provides both practical assistance and emotional reassurance, helping YPLHIV and their guardians ART access and adherence.

***The continuous education about access to and adherence to ART from health workers in the HIV clinics:*** A female respondent recruited in Y HIV clinic said: “At times when they feel that your adherence to viral suppression is changelling, they [HIV clinic staff] counsel you ensure that you are ok and do not feel discouraged.” A child guardian from the same clinic reported: “The service provision is good because the doctors have customer care and communication skills … they listen to you and they help you.” Similarly, a female recruited from the same HIV clinc stated: “They give us advice on many issues so that we feel peace and hope. For sure, we have no doubt about the staff here because they help us fully even when we face challenges.” A guardian from the same clinic also said: “If the ART is finished, we just come and take another one. For me, there are no challenges” (R1, guardian, Y HIV clinic). Finally, a child guardian from X clinic explained that: “The services here are good and I am grateful when they remind us because, personally, my memory is poor.”

These quotes, shared by YPLHIV and their guardians, highlight the essential role of health workers in HIV clinics. Their support is provided through education, counseling, and interpersonal assistance, helping young people and their guardians understand the importance of treatment adherence and retention in care. Their ability to listen, offer practical assistance, and provide appointment reminders fosters trust, reduces barriers to ART accessing, and ensures care continuity.

***The continuous counselling from social workers in the community:*** Especially for young people, education by community health workers and clubs is a community resource to early testing and motivate them to adhere to ART. A female on ART at one of the clinics reported that: “I would like community health workers to go into the communities and educate the society. Even though young people are afraid, once they are counseled and informed about what to do, they regain hope and will want to continue using the ART.” (R4, female, Y HIV clinic). Another female respondent said that: “There was a time when I lost hope and said I was not taking my ART anymore. But I remember there were community health workers who came to my house privately. They said they wanted to talk to me. I felt at peace and wanted to continue with the ART, until I was well.” (R4, female, HIV clinic). In addition, another female respondent participant stated that: “At times when they feel that your adherence to viral suppression is changelling, they (HIV clinic staff) counsel you such that they make sure you are ok and do not feel discouraged.” (R3, female, Y HIV clinic).

***The continuous guidance and encouragement from peers in health/social clubs:*** A child guardian from X clinic recognised the role of education clubs, saying that: “The potential of the club is to be a place to meet and compare, from which knowledge is gained. These clubs are helpful for these children, especially girls entering adolescence. They explain the reasons for taking ART and be told to stop un-protected sexual intercourse.” Similarly, a male taking care from the same clinic also said: “The club used to support and encourage us and make us feel like we are part of society. They used to build us up and teach us. Even those who had given up regained hope through such clubs.” Finally, a female respondent from another clinic (Y) reported: “These clubs are useful because when you have lost hope, a person like you, the same age and so confident, comes along. You feel encouraged. These clubs are useful and should be further promoted on the streets.”

These quotes show that educational clubs, supported by HIV-positive peer educators of the same age, provide YPLHIV knowledge, risky sexual behaviors, and ART adherence. Peer support is particularly effective in motivating and educating YPLHIV, as peers serve as relatable role models, fostering hope and improving engagement in treatment.

***Having a watch and sticking to the usual time of swallowing medicine:*** A female respondent recruited at Y HIV clinc, said: “I have two kinds of ART. I take one form at 09:00 in the morning and another at 21:00 at night.” A child guardian form Z clinic stated: “My child takes ART every day 8:00 a.m.” A young male respondent receiving treatment at X HIV clinic affirmed: “My watch reminds me that it’s time. I put the watch on my study table, and I take my ART at 9:00 p.m. and them go to sleep.” A guardian from the same clinic stated: “I always use a phone to keep track of time and remind him to take ART at 08:00.” Another guardian respondent from different clinic (Y) reported: “We use a phone to check the time.”

These quotes be useful to clarify that establishing routines, using reminders and having treatment supporters are effective strategies that help adolescents and children, as well as young adults, adhere to ART. Tools, such as phones and watches, serve as practical aids, supporting consistent medication-taking and promoting treatment continuity.

***Receiving regular ART reminders through the telephone or television from peers***,*** tutors***,*** caregivers and significant others enhance adherence to ART:*** A child guardian from X clinic explained: “What reminds him is a television program that he loves and always watches. So when it starts, he remembers that it’s time to take ART.” A male respondent from different clinic (Y) said: “ I shared my challenges with my sister. She advises me very well. I am grateful to her because she helps me to solve some of my challenges.”

These quotes highlight how regular reminders from phones, television programs or support figures, such as family members/guardian and peers, help YPLHIV adhere to ART. These reminders ensure timely medication intake, provide encouragement and practical guidance, positively impacting treatment adherence.

## Discussion

This study aimed to identify the factors that hinder and facilitate YPLHIV’s access to and adherence to ART.

Perceived stigmatizing and discriminatory attitudes towards YPLHIV were identified as the primary barriers. The literature highlights a negative correlation between HIV-related stigma and the health and well-being of YPLHIV, which adversely affects their care, treatment adherence and overall quality of life. So their mitigation constitutes a priority and represents a significant public health challenge [13, 14]. This study found that stigmatizing and discriminatory attitudes from family members towards hildren living with HIV can undermined their sense of family belonging, diminished self-esteem, and contributed to apathy, depressive symptoms, anxiety and suicidal tendencies. Notably, our findings highlighted that discrimination and social isolation related to HIV status were prevalent in conflictual family environment, often characterized by the presence of many children and co-wives. Such a context leads to demotivation and represents a significant barrier to ART adherence. Similarly, the stigmatizing and discriminatory attitude from neighbors, friends, schoolmates, teachers and even healthcare workers, contributes to weakening their motivation to adhere ART. From the perspective of young people, the need to keep their serostatus secret is central to the extent to which they are willing to stop taking ART in order not to be seen to be taking it [15].

The unwanted chats about the young people’s personal HIV situation by service providers and the need for privacy were the reasons for discontinuation of ART. To avoid disclosing their HIV status, young people may stop or avoid participation in HIV clinics and support activities such as clubs [13, 15, 16].

Like in other earlier studies [13], this study also showed that the guardians do not usually disclose their children’s HIV status to protect them from being stigmatized and discriminated by the family and community members. They often take on the role of reminding their children to obtain and adhere to their ART, except in cases where they are unable to consistently support their health needs. In such instance, they assign a trustworthy family member the responsibility, hence underscoring the importance of sharing this responsibility with other family members [14].

This study identified additional factors hindering ART access and adherence.

Specifically, YPLHIV frequently forgot to take the antiretroviral medications due to busy schedules which often included household chores, schoolwork, and social events. Consistent with other research, our findings also indicate that engaging family members as antiretroviral therapy supporters helps to remind and motivate YPLHIV to adhere to their ART regimen [14]. This study also showed that some structural factors deter YPLHIV from accessing and adhering to ART. Poverty caused food insecurity, which deterred some YPLHIV from taking their medications. Additionally, the long distances to HIV clinics, associated costs, travel time, and weather-related travel difficulties further impede consistent access to treatment. A previous study showed that the extensive time required to access services is a factor hindering ART adherence. Men often prioritize activities to support their families, so when clinic schedule conflict with these responsibilities, ART adherence declined [16].

Various factors facilitate ART access and adherence, as shown by both our findings and those of previous studies. In particular, emotional and practical support from close contacts was a fundamental factor in helping YPLHIV learn to live with the infection, access health and community services, adhere to ART, and cope with daily stigma. Family members, friends, health and social service workers are therefore key figures in supporting YPLHIV and in raising awareness among service providers and the community at large [14, 15]. Schools also play a key role in promoting an inclusive, stigma-free environment. Young people spend a significant amount of time in educational institutions, so it is important that they experience support and inclusion there. This facilitates acceptance of their HIV status and ART adherence. For this reason, it is essential that teachers possess the necessary skills and knowledge in sexuality education and reproductive health rights to promote knowledge and implement effective infection prevention strategies [13].

As reported in other studies, our results showed that peer educators played a valuable role, not only in providing educational and emotional support, but also in facilitating the transition from personal denial to collective advocacy and from HIV diagnosis to personal adherence to ART. Observing the positive outcomes achieved by individuals with similar health conditions, such as longer survival and more economic productivity, appears to have motivated YPLHIV to continue with antiretroviral therapy on their own [16]. Furthermore, the establishment of HIV clubs in schools and the community as well as the sensitisation of communities about stigma, could help to provide peer support and ensure social inclusion of YPLHIV. This could in turn create supportive environments for access and adherence to ART. Also, HIV clinics, youth-focused HIV services and community health workers are crucial in promoting awareness about high-risk behaviour, early testing for HIV and enrollment and adherence to ART. This can be achieved through developing and implementing Social and Behavioural Change (SBC) strategies, which promote awareness and behavioural change within communities. This is particularly relevant in the context of the current advocacy for U = U (Undetectable = Untransmittable) messaging and the assurance provided to people living with HIV that they can lead a successful life, comparable to their counterparts without the disease, if they adhere to their ART. Clinics should become more youth-friendly environments for YPLHIV. Studies have shown that addressing organizational barriers to healthcare, such as long waiting time at HIV clinics, is effective in improving access and adherence to ART [13]. This would include harmonising the workdays and time for HIV clinics with the needs of young people to enable them ART access at their convenience. HIV clinics and adolescent friendly services should be promoted at facility level by ensuring that youth service delivery points are confidential and adequately staffed with experts in the delivery of adolescent-friendly approaches [17]. In addition, the index, moonlight and social network testing methods represent the optimal way of ensuring early diagnosis and, should be scaled up to enhance treatment outcomes for adolescents and youth living with HIV.

### Limitations

Several limitations emerged at different stages of this study. The first key limitation was related to language barriers, as many participants spoke only the local Sukuma language. Since the study and data collection instruments were originally developed in English, a language not understood by the participants, it was necessary to recruit bilingual research assistants to facilitate communication. These assistants translated the interview and focus group questions from English into Sukuma and translated participant responses back into English. However, this process introduced the potential misinterpretation bias, including misrepresentation of participant meaning or the loss of important semantic nuances, which could have biased the findings. To mitigate this risk, each sentence was *verbatim* transcribed, with both the English and translated the original Sukuma presented side by side to allow for consistency checks and quality control.

Secondly, as is the case for most qualitative research, this study recruited and interviewed a few participants from three HIV clinics in the Shinyanga area. Therefore, the findings may not be generalizable to YPLHIV in other parts of Tanzania.

In addition, while in-depth interviews were conducted to further explore themes that emerged during the focus group discussions, the IDIs largely confirmed the same patterns without adding substantially new insights. This may suggest a saturation of themes, but also limits the breadth of new perspectives captured beyond the group context.

## Conclusion

The effectiveness of antiretroviral therapy depends on early initiation, strict adherence to prescribed dosing schedules, and regular clinical monitoring to minimize the risk of drug resistance and other negative treatment outcomes. This study highlighted that, while many young people living with HIV (YPLHIV) on antiretroviral therapy (ART) do not consistently adhere to their treatment due to negative experiences such as stigma, discrimination, concerns about disclosing their HIV status, and structural barriers, a significant proportion manage to maintain adherence thanks to critical protective and supportive factors, including family and community support and privacy in HIV clinics. These findings strongly align with Bronfenbrenner’s social ecological model [9], which posits that adolescent behavior is shaped by the interaction of biological, psychological, and familial characteristics, community and school values, societal and cultural norms, and changes in public policy and living conditions [10, 18]. The results underscore the urgent need for comprehensive, multi-level interventions targeting personal, interpersonal, community, and systemic barriers to ART adherence, in order to improve health outcomes and overall well-being for young people in the Shinyanga region.

### Importance and Implications of the Study

The results of this research call for the integration of mental health services into the continuum of HIV care; the continuous provision of counselling related to disclosure of the HIV status and enrollment and adherence to ART among young people receiving ART at the family, school, community and clinic levels; and the scaling-up of the identified factors which support access/adherence to ART throughout the care pathway [10, 19].

## Data Availability

The underlying data for this study are available from the corresponding author upon reasonable request.

## Declarations

### Institutional Research Board Approval

Ethical approval was granted by the Tanzania National Institute for Medical Research in November 2022 (NIMR/HQ/R.8a/Vol.IX/4162). The study was conducted in accordance with the ethical principles outlined in the Declaration of Helsinki and The International Conference on Harmonization Good Clinical Practice guidelines.
